# Phase separating Rho: a widespread regulatory function of disordered regions in proteins revealed in bacteria

**DOI:** 10.1038/s41392-023-01505-5

**Published:** 2023-06-21

**Authors:** Eric Schumbera, Pablo Mier, Miguel A. Andrade-Navarro

**Affiliations:** Institute of Organismic and Molecular Evolution, Faculty of Biology, Johannes Gutenberg University of Mainz, 55128 Mainz, Germany

**Keywords:** Cell biology, Molecular biology, Computational biology and bioinformatics

A recent study published in *Science* by Krypotou et al. reported the role of an intrinsically disordered region (IDR) in the transcription termination Rho protein from *Bacteroides thetaiotaomicron*: in response to starvation Rho phase separated, which triggered an increase in Rho activity and expression changes.^1^ These results demonstrate that a highly conserved protein can acquire new regulatory properties in evolution by gaining an IDR that leads to protein phase separation.

Rho is a protein that binds RNA to terminate transcription under certain conditions, and it is, therefore, a central regulator of gene expression being widely present in bacteria save for a few taxa, such as Cyanobacteria, Mollicutes and several Firmicutes.^2^ This latter work noted the variability in the length of Rho, explained by a largely variable insertion domain situated between Rho’s N-terminal helix bundle (NHB) and cold shock domain (CSD)-like RNA binding regions, which included R/K-rich regions in Actinobacteria and Q/N-rich in Bacteroidetes. This is the IDR studied by Krypotou et al., who also noted that the *B. thetaiotaomicron* IDR contains two compositionally biased regions: an E/K-rich region and a Q/N-rich region surrounding a small conserved element.

Amino acid regions biased for one or two amino acids are very frequent (constituting about 1% of residues in Eukarya and 0.7% in other taxa, respectively^3^). They confer structural and evolutionary properties to proteins very different from those of regions of higher complexity that result in globular domains. Their frequency makes studying their function an important task, but this is complicated by their evolutionary and compositional variability. Functional involvement in phase separation of IDRs containing regions of low complexity has been hinted at because compositionally biased regions occur more often in IDRs of proteins involved in liquid–liquid phase separation (LLPS).^4^ Phase separation is receiving increasing attention as a mechanism by which cells can generate macromolecular interactions less stable than stoichiometric complexes.^5^

The paper of Krypotou et al. illustrated the rich variability of compositions in the IDRs of Rho with a set of 27 proteins (Supplementary Fig. S1 in^1^). In just these few examples, the variability of compositions and repeats is staggering (Fig. 1a), but a long IDR is by no means required for Rho function, as exemplified by the proteins from *Escherichia coli*, *Lact. sp*. (actually, *Loigolactobacillus coryniformis*), and a few other Firmicutes, which have almost no residues between the NHB and the CSD-like domains.

**Fig. 1:**
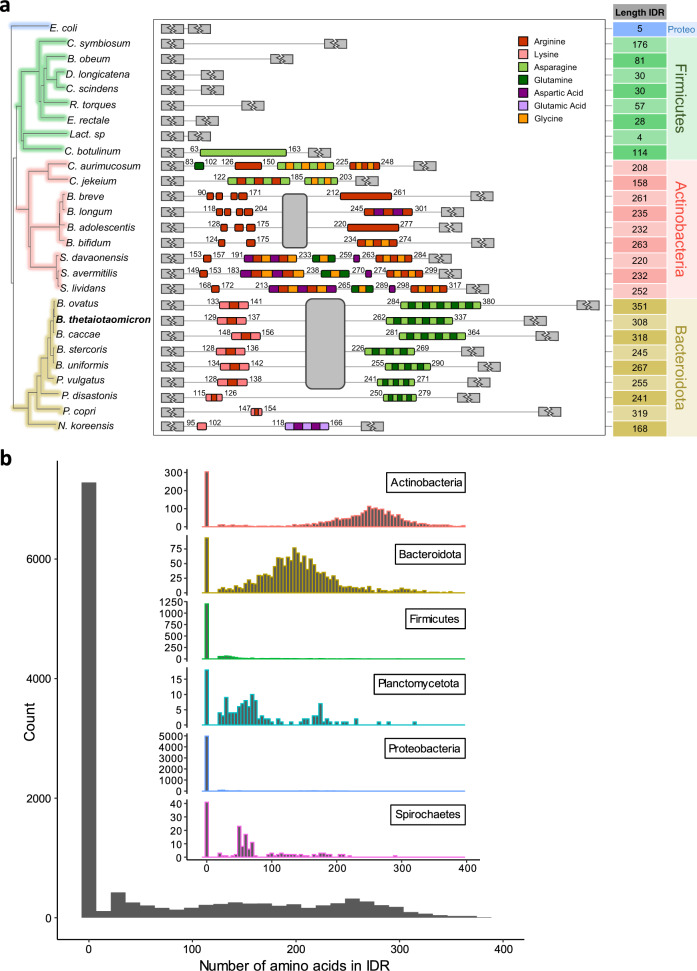
Properties of IDRs in Rho proteins of bacteria. **a** Amino acid biased regions of Rho IDRs grouped by taxonomy. Lines indicate disordered sequences. Coloured boxes on them indicate composition-biased regions colour coded by amino acid (see legend; obtained following methods described in Ref. ^3^ and manually curated). The two large grey boxes indicate conserved motifs of complex composition present in *Bifidobacterium* (Actinobacteria) and in *Bacteroides* (Bacteroidota). The 27 sequences used are those in Supplementary Fig. S1 of ref. .^1^ **b** Distribution of amino acids predicted to be in IDRs (by UniProtKB release 2023_01) in Rho proteins from 12,228 bacterial species (one protein per species was chosen). The inset shows values for Actinobacteria (*n* = 2366), Bacteroidota (1476), Firmicutes (1738), Planctomycetota (135), Proteobacteria (5461) and Spirochaetes (158). The set of 27 sequences in panel a contains only one Proteobacteria (*E. coli*) and none of Planctomycetota or Spirochaetes

It can be observed that IDRs from taxonomically related species have similar compositionally biased regions of one, two or three amino acids (coloured boxes in Fig. 1a). The amino acid types observed include arginine, asparagine, aspartic acid and glycine, which are four of the six amino acid types more frequently observed in compositionally biased regions contained within longer IDRs in human proteins.^4^

Some of the most complex IDRs possess short tandem repeats of a few amino acids. For example, while Rho sequences lack IDRs in most Firmicutes, two Lachnospiraceae (spore-forming bacteria that ferment plant polysaccharides and are abundant in gut microbiota) display IDRs with tandem repeats: 14 units with consensus sequence YQSRGD in *Clostridium symbiosum* or 5 units with consensus sequence RNYQD in *Blautia obeum*. These cases reflect independent events of sequence tandem duplication converging to repeats that contain YQRD.

This variability of lengths and composition suggests that bacteria adapt their use of IDRs in LLPS through sequence variations. How frequently? To answer this question, we obtained one Rho sequence from each of the 12,228 bacteria and observed the distribution of the number of residues predicted to be in IDRs (Fig. 1b). Results confirmed that having an IDR in Rho is not the norm (4947 sequences, 40.5%, lack IDRs). Interestingly, the results were very different by taxa, suggesting different amounts of residues in IDRs for Actinobacteria, Bacteroidota, Planctomycetota and Spirochaetes, and the absence of IDRs in most Firmicutes and Proteobacteria (Fig. 1b, inset).

Taken together, the variability and widespread use of IDRs in Rho suggest that LLPS formation would occur in a majority of bacterial species. Given the diversity of needs and environments of the considered bacteria and lacking further experimental work on the IDRs of other species, we hypothesise that the function of IDRs in Rho is not necessarily to react to starvation but more generally to synchronise Rho proteins activity to different environmental cues. The different types of compositional bias that we found are incredibly rich, and their conservation at certain taxonomic levels probably reflects a different functional tuning of Rho at each of them. These differences could be related to different evolutionary pressure. In our wide analysis of IDRs in Rho (Fig. 1b), we did not find a particular association with bacterial structural properties or pathogenicity. But, when looking at the distribution of Rho proteins in bacteria, D’Heygere et al.^2^ noted that species lacking Rho have smaller genomes, which is an indication of bacteria with parasitic or symbiotic lifestyle living in stable conditions. They interpreted this as an indication that selective pressure for Rho functionality might be related to bacteria living in a complex ecosystem and needing to adapt to environmental changes. Since the inclusion of an IDR in Rho expands its functionality, we would expect that similar evolutionary pressure also drives this. These analyses support the notion that Krypotou et al. has revealed a role of disordered regions in enriching the regulatory and environmental sensing abilities of a conserved protein; this function is widespread and is based on an array of IDRs with a staggering variation in lengths and composition, uncovering a universe of functional possibilities for IDRs in just this one protein family.

Establishing the correspondence between sequence composition variations and function in compositionally biased regions of IDRs is a challenge ahead. We expect that the work of Krypotou *et al*. will inspire further research providing increasing support for the function of IDRs and their compositionally biased regions for RNA binding proteins, with IDRs in particular, but also in general for other proteins bearing IDRs that could be generating complex hubs for regulatory interactions in many different contexts. This should facilitate the characterisation of the elusive IDR functionality, advancing our knowledge of protein function.

## References

[1] Krypotou E (2023). Bacteria require phase separation for fitness in the mammalian gut. Science.

[2] D’Heygère F (2013). Phyletic distribution and conservation of the bacterial transcription termination factor Rho. Microbiology.

[3] Mier P (2022). Regions with two amino acids in protein sequences: a step forward from homorepeats into the low complexity landscape. Comp. Struct. Biotechnol. J..

[4] Kastano K (2022). Functional tuning of intrinsically disordered regions in human proteins by composition bias. Biomolecules.

[5] Bergeron-Sandoval LP (2016). Mechanisms and consequences of macromolecular phase separation. Cell.

